# Antimicrobial activity of mul-1867, a novel antimicrobial compound, against multidrug-resistant *Pseudomonas aeruginosa*

**DOI:** 10.1186/s12941-016-0134-4

**Published:** 2016-03-22

**Authors:** George Tetz, Daria Vikina, Victor Tetz

**Affiliations:** TGV-Laboratories LLC, 303 5th avenue, # 2012, New York, NY 10016 USA; Institute of Human Microbiology, New York, NY 10016 USA

**Keywords:** Biofilms, Mucoid, Non-mucoid, Multidrug-resistant, *P. aeruginosa*, Cystic fibrosis

## Abstract

**Background:**

There is an urgent need for new antimicrobial compounds to treat various lung infections caused by multidrug-resistant *Pseudomonas aeruginosa* (MDR-PA).

**Methods:**

We studied the potency of Mul-1867 against MDR-PA isolates from patients with cystic fibrosis, chronic obstructive pulmonary disease, and ventilator-associated pneumonia. The minimal inhibitory concentrations (MICs) and minimum biofilm eliminating concentrations (MBECs), defined as the concentrations of drug that kill 50 % (MBEC_50_), 90 % (MBEC_90_), and 100 % (MBEC_100_) of the bacteria in preformed biofilms, were determined by using the broth macrodilution method.

**Results:**

Mul-1867 exhibited significant activity against MDR-PA and susceptible control strains, with MICs ranging from 1.0 to 8.0 µg/mL. Mul-1867 also possesses anti-biofilm activity against mucoid and non-mucoid 24-h-old MDR-PA biofilms. The MBEC_50_ value was equal to onefold the MIC. The MBEC_90_ value ranged from two to fourfold the MIC. Moreover, Mul-1867 completely eradicated mature biofilms at the concentrations tested, with MBEC_100_ values ranging between 16- and 32-fold the MIC. Mul-1867 was non-toxic to Madin-Darby canine kidney (MDCK) cells at concentrations up to 256 µg/mL.

**Conclusion:**

Overall, these data indicate that Mul-1867 is a promising locally acting antimicrobial for the treatment and prevention of *P. aeruginosa* infections.

## Background

*Pseudomonas aeruginosa* is an important pathogen contributing to lung infections in patients with pulmonary diseases such as cystic fibrosis (CF), chronic obstructive pulmonary disease (COPD), and ventilator-associated pneumonia (VAP) [1–3]. By 21 years of age, most CF patients are chronically infected with *P. aeruginosa* that, once established, is difficult to eradicate and is associated with major morbidity [4–6]. *P. aeruginosa* is also an important contributor to acute exacerbations in COPD and VAP [7]. Some *P. aeruginosa* strains are mucoid due to overexpression of alginate, and are less susceptible to the host immune system [8]. Overproduction of alginate, and conversion of *P. aeruginosa* to a mucoid phenotype, are considered markers of chronic lung infections and indicate a decreased life expectancy and a more rapid decline in pulmonary function [9].

Both mucoid and non-mucoid strains of *P. aeruginosa* form biofilms that are associated with persistence in chronic infections, and also contribute to bacterial colonization in acute infections [10]. Established biofilms display high resistance to antimicrobials, and finding ways to disrupt these films is a critical goal in combatting these infections [11, 12]. *P. aeruginosa* survival in biofilms may be determined by multiple factors including the surface film and the presence of extracellular polymeric substances [13, 14]. Moreover, clinical isolates of multidrug resistant *P. aeruginosa* (MDR-PA) are being observed with increasing frequency [15]. Thus, biofilms formed by MDR-PA are a serious and often fatal problem for patients with lung infections, and many antibiotics are ineffective against bacteria in biofilm communities at concentrations of 10–1000 times the expected minimum inhibitory concentration (MIC) [16].

During the last decade, it became evident that existing antibiotics were unable to treat respiratory infections caused by *P. aeruginosa* effectively [17]. Lung infections caused by MDR-PA were poorly responsive even to nebulized therapy when the aerosolized antibiotics achieved high concentrations directly at the pathological site [18]. It was also shown that many antibiotics, even when able to decrease the number of live bacteria within the biofilm, could not completely eradicate preformed biofilms [19].

**Fig. 1:**
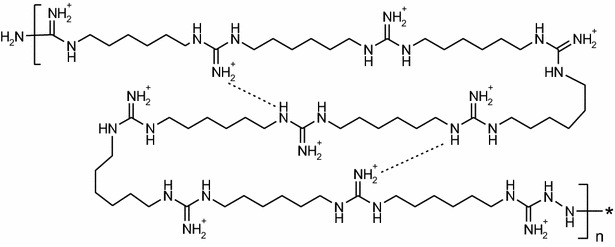
Chemical structure of Mul-1867 n = 1–20

The poor therapeutic results with existing antibiotics revealed a critical need for the development of new antimicrobials active against MDR-PA that can be used to treat lung infections. The goal of this study was to examine in vitro MICs and minimal biofilm eradication concentrations (MBECs) of the novel antimicrobial compound, Mul-1867 (poly-N,N’-hexamethyleneguanidine-poly-N1,N4-hexamethyleneaminoguanidine; Fig. 1). The mechanism of action of Mul-1867 involves a nonspecific attack on the cell walls of MDR-PA isolated from patients with respiratory tract infections [20]. Furthermore, the anti-biofilm efficacy of Mul-1867 against preformed mucoid and non-mucoid 24-h-old biofilms of MDR-PA was assessed.

## Methods

### Test substance and antibiotics

Mul-1867 (Fig. 1) was synthesized by TGV-Therapeutics (New York, NY, USA).

The antibiotics used for these studies included amikacin, aztreonam, ceftazidime, meropenem, piperacillin, and tobramycin, which are currently administered to treat lung infections in patients with CF, COPD, or VAP. These antibiotics were purchased from Sigma-Aldrich (St. Louis, MO, USA).

### Bacterial strains and growth conditions

MDR-PA isolates from CF patients including the AGR14, MR45, and VT-CF-234 strains, as well as one susceptible isolate, SEV 7; were obtained from the CF Foundation Therapeutics Development Network Resource Center for Microbiology at Seattle Children’s Hospital (Seattle, WA, USA) and from the Institute of Human Microbiology, LLC (DE, USA). MDR-PA isolates from COPD and VAP patients, VT-COPD-274 and VT-VAP-72, respectively, were supplied by the Institute of Human Microbiology. The three strains classified phenotypically as mucoid were SEV 7, VT-CF-234, and VT-COPD-274. The strain used for quality control of the antibiotic panels was *P. aeruginosa* ATCC 27853 from the American Type Culture Collection (Manassas, VA, USA).

All bacterial strains were subcultured from freezer stocks onto Mueller–Hinton agar plates (Oxoid Ltd., London, England) and incubated at 37 °C overnight. All subsequent liquid subcultures were derived from colonies isolated from these plates and were grown in Mueller–Hinton broth (MHB) (Oxoid). The isolates were categorized as susceptible, intermediate, or resistant according to the Clinical and Laboratory Standards Institute (CLSI) breakpoints guidelines (Table 1) [21].

**Table 1:** Bactericidal activities of Mul-1867 and other antibacterials against non-mucoid *P. aeruginosa* isolates

Antibacterials	Susceptibility breakpoint (µg/mL)	*P. aeruginosa* strain minimal inhibitory concentration (µg/mL)			
		AGR14	MR 45	VT-VAP-72	ATCC 27853
Mul-1867	ND^a^	8	8	4	1
Amikacin	≤16	128	256	128	4
Aztreonam	≤8	32	128	64	2
Ceftazidime	≤8	32	32	16	2
Meropenem	≤2	32	64	32	1
Piperacillin	≤16	>256	256	128	4
Tobramycin	≤4	32	32	32	1

^a^ *ND* no data

### In vitro antimicrobial susceptibility testing

The MICs for antimicrobials were determined by the broth macrodilution method according to CLSI guideline [22, 23]. A standard bacterial inoculum of 5 × 10^5^ colony forming units (CFU)/mL was used. Serial twofold dilutions of the antimicrobials were prepared in cation-adjusted MHB. The MIC was defined as the lowest concentration of antibiotic that completely inhibited visible growth. Experiments were conducted in triplicate.

### Effect of Mul-1867 against *P. aeruginosa* biofilms

In each well of a 96-well flat-bottom polystyrene tissue culture microtiter plate (Sarstedt, Numbrecht, Germany), 200 μL of a standardized *P. aeruginosa* inoculum (5 × 10^5^ CFU/mL) in cation-adjusted MHB were added. Following a 24 h incubation period at 37 °C, biofilm samples were washed twice with phosphate-buffered saline to remove non-adherent bacteria and then exposed for 24 h to 200 μL of MHB containing Mul-1867 at 1-, 2-, 4-, 8-, 16-, 32-, and 64-fold the MIC. Untreated biofilms were used as the negative controls. After the exposure, well contents were aspirated to prevent antimicrobial carryover and each well was washed three times with sterile deionized water.

To estimate the CFU number, biofilms were scraped thoroughly, with particular attention paid to the well edges [11]. The well contents were aspirated, placed in 1 mL of isotonic phosphate buffer (0.15 M, pH 7.2), and the total CFU number was determined by serial dilution and plating on Mueller-Hinton agar.

The MBECs were determined as the concentrations of drug that killed 50 % (MBEC_50_), 90 % (MBEC_90_), and 100 % (MBEC_100_) of the bacteria in preformed biofilms compared to the untreated control (100 % viability). MBEC sensitivities were determined using the 2012 Clinical and Laboratory Standards Institute guidelines for interpretation [23]. Tests were performed in triplicate on three separate occasions, and the results were averaged.

### Cytotoxicity assay

Cytotoxicity of Mul-1867 on Madin-Darby canine kidney (MDCK) cells was determined by the microculture tetrazolium (MTT) assay. Briefly, cells (1 × 10^5^/well) were plated in 100 μL of minimal essential media in 96-well plates and incubated overnight in a 5 % CO_2_ atmosphere. Serial dilutions of Mul-1867, with final concentrations corresponding to a range of 8–256 µg/mL, were added to the cells that were then incubated at 37 °C for a further 24 h. Control wells were treated with sterile water. MTT analysis was then performed based on the standard method [24].

After incubation, an aliquot of [3-(4,5-Dimethylthiazol-2-yl)-2,5-diphenyltetrazolium bromide] solution (Sigma-Aldrich, St. Louis, MO, USA) at 0.5 mg/mL was added to each well, and following a further 30 min incubation at 37 °C, the medium was discarded and the formazan blue formed in the cells was dissolved with 100 μL of dimethyl sulfoxide. Absorbance at 570 nm was determined with a microplate reader (Stat-Fax-2100, Awareness Technology Inc, USA). The absorbance of the formazan formed in non-treated cells was considered 100 % viability.

### Statistical analyses

Results of the cytotoxicity assay are presented as mean ± standard deviation of three independent experiments. All statistical analyses were performed using the statistics package Statistica for Windows (version 5.0). Results are reported as the mean ± standard error for each group. Non-parametric paired Wilcoxon signed rank test was applied to analyse pre- and post-challenge differences. P < 0.05 was considered significant.

## Results

### In vitro antimicrobial susceptibility testing of *P. aeruginosa* isolates

The MICs of Mul-1867 and other antibiotics against non-mucoid *P. aeruginosa* isolates are shown in Table 1.

Mul-1867 exhibited significant activity against all isolates that were resistant to amikacin, aztreonam, ceftazidime, meropenem, piperacillin, and tobramycin according to CLSI criteria, as well as against susceptible and control strains.

Of the seven MDR-PA isolates, three had the mucoid phenotype that is widespread among CF patients and is associated with reduced success of treatment. Mul-1867 exhibited a high level of antimicrobial activity against mucoid MDR-PA strains irrespective of their resistance to other antibiotics (Table 2) [25, 26].

**Table 2:** Minimal inhibitory concentrations of Mul-1867 and other antibacterials against mucoid *P. aeruginosa* isolates

Antibacterials	Susceptibility breakpoint (µg/mL)	*P. aeruginosa* strain minimal inhibitory concentration (µg/ml)		
		SEV 7	VT-CF-234	VT-COPD-274
Mul-1867	ND^a^	2	2	4
Amikacin	≤16	128	64	256
Aztreonam	≤8	64	64	128
Ceftazidime	≤8	2	32	32
Piperacillin	≤16	2	64	128
Meropenem	≤2	32	8	16
Tobramycin	≤4	16	128	64

^a^ *ND* No data

There was no statistically significant difference between the activity of Mul-1867 against the mucoid vs. non-mucoid *P. aeruginosa* strains. The MIC values ranged from 1 to 8 µg/mL for the non-mucoid strains, and from 2 to 4 µg/mL for the mucoid strains.

### Effect of Mul-1867 against preformed *P. aeruginosa* biofilms

The anti-biofilm activity of Mul-1867 against 24-h-old *P. aeruginosa* biofilms was analyzed to reveal possible differences in susceptibility patterns between non-mucoid and mucoid *P. aeruginosa* strains. The activity of Mul-1867 on mature biofilms, tested at concentrations equal to a multiple of the MIC, is summarized in Table 3. After 24 h of growth, the CFUs of *P. aeruginosa* AGR14 and *P. aeruginosa* VT-CF-234 biofilms were 1.8 × 10^8^ CFU/mL and 3.5 × 10^8^ CFU/mL, respectively.

**Table 3:** Susceptibility results of Mul-1867 against *P. aeruginosa* strains

Isolate	Mucoid	MBEC_50_ (µg/mL)	MBEC_90_ (µg/mL)	MBEC_100_ (µg/mL)
*P. aeruginosa* AGR14	No	8	32	256
*P. aeruginosa* VT-CF-234	Yes	2	4	32

*MBEC* minimum biofilm eliminating concentrations

A comparison between the MIC and MBEC values indicated that Mul-1867 was a highly active antimicrobial exhibiting dose-dependent effects against the *P. aeruginosa* strains tested. Mul-1867 showed MBEC_50_ and MBEC_90_ values of 1- and 4-fold the MIC, respectively, against a non-mucoid strain. Mul-1867 displayed comparable activity against a mucoid strain with MBEC_50_ and MBEC_90_ values of 1- and twofold the MIC, respectively.

Reduction in cell numbers by 90 % suggested that Mul-1867 has the potential for biofilm eradication, which is an important goal in antimicrobial treatment. Thus, we determined the MBEC_100_ after treatment of *P. aeruginosa* biofilms with Mul-1867 for 24 h.

In this assay, Mul-1867 completely eradicated mature *P. aeruginosa* biofilms at the concentrations tested, with MBEC_100_ values of 32- and 16-fold the MIC for the AGR14 and VT-CF-234 strains, respectively. The ability to eradicate a biofilm at a concentration eightfold higher than that required to reduce biofilm bacteria by 90 % suggested that Mul-1867 possesses significant anti-biofilm activity.

### Cell cytotoxicity of Mul-1867

**Fig. 2:**
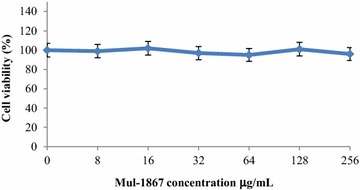
Cytotoxic effect of Mul-1867 on Madin-Darby canine kidney cell lines as determined by the microculture tetrazolium assay

The compound did not exhibit any statistically significant cytotoxicity against MDCK cells at concentrations ranging from 8 to 256 µg/mL, as tested by the MTT assay 24 h after challenging with Mul-1867 (Fig. 2).

## Discussion

*P. aeruginosa* presents substantial problems for patients with CF, and is a major predictor of morbidity [27]. For the last decade, the emergence of MDR-PA strains has posed treatment challenges in patients with COPD and VAP [28]. In the current study, we found that a novel drug candidate, Mul-1867, exhibits a high level of antimicrobial activity against both MDR and susceptible clinical isolates of *P. aeruginosa* from patients with life-threatening lung infections.

We showed previously that Mul-1867 is a fast-acting antimicrobial whose mechanism of action involves a nonspecific attack on the bacterial cell wall that causes leakage of cell contents [20]. Mucoid strains lead to the persistence of *P. aeruginosa* infections. The data available in the literature concerning the effect of mucoidity on antimicrobial susceptibility show some differences depending on the type of antibiotic being tested [29]. Our data indicate that there is no cross-resistance between amikacin, aztreonam, ceftazidime, meropenem, piperacillin, and tobramycin vs. Mul-1867 against susceptible and MDR-PA strains tested in this study.

*P. aeruginosa* biofilms respond poorly to treatment with existing medicines due to the presence of additional barriers including a surface film or extracellular polymeric substances that reduce antibiotic penetration [30]. Biofilms formed by MDR-PA are almost completely insensitive to treatment with antibiotics that have high MBEC values that cannot be achieved at the site of infection using currently recommended dosages, or even with local administration directly to the site of infection [31]. There are also reports that biofilms formed by some strains of *P. aeruginosa* cannot be eradicated at any concentration of antibiotics that are commonly used for therapy. Importantly, however, Mul-1867 was equally effective against mucoid and non-mucoid *P. aeruginosa* strains.

The MBEC_90_/MIC ratio is an important parameter for choosing the best antibacterial to treat *P. aeruginosa* biofilm-associated infections (lower is better). Our studies revealed that Mul-1867 possessed low MBEC_90_/MIC ratios (between two and four). Moreover, biofilms formed by both non-mucoid and mucoid *P. aeruginosa* strains were totally eradicated with Mul-1867 at concentrations of 32- and 16-fold the MIC, respectively.

Positively charged antibiotics bind to extracellular polymeric substance and extracellular DNA, which decreases their bioavailability [30]. Mul-1867 is a positively charged molecule, and its antimicrobial activity was also reduced by the biofilm’s components. However, its activity level remained high enough to achieve total eradication of biofilms.

Cell viability assessed by the MTT assay revealed that Mul-1867 did not display cytotoxic effects at the tested concentrations (between 8 to 256 µg/mL). The absence of apparent toxicity to MDCK cells at the highest concentration of Mul-1867 that was used against the least sensitive *P. aeruginosa* strain tested in this study (AGR14) is a notable attribute of this topical antimicrobial compound.

The high levels of Mul-1867 activity against the clinical isolates tested in this study are consistent with those found in our previous work [20]. This demonstrates the broad antimicrobial activity of this molecule against planktonic and sessile gram-positive and gram-negative bacteria. The efficacy of Mul-1867 against both non-mucoid and mucoid strains of *P. aeruginosa* raises the possibility that it may serve as a locally acting antimicrobial compound and may be able to successfully eradicate biofilm infections caused by *P. aeruginosa*.

## Abbreviations

CF: cystic fibrosis
CFU: colony forming units
CLSI: Clinical and Laboratory Standards Institute
COPD: chronic obstructive pulmonary disease
MBEC: minimum biofilm eliminating concentrations
MDCK: madin-Darby canine kidney
MDR-PA: multidrug-resistant *Pseudomonas aeruginosa*
MHB: Mueller–Hinton broth
MIC: minimal inhibitory concentration
VAP: ventilator-associated pneumonia
